# Field investigation combined with modeling uncovers the ecological heterogeneity of *Aedes albopictus* habitats for strategically improving systematic management during urbanization

**DOI:** 10.1186/s13071-023-05926-7

**Published:** 2023-10-25

**Authors:** Xiang Guo, Lei Luo, Yuxiang Long, Pingying Teng, Yuehong Wei, Tian Xie, Li Li, Qingqing Yin, Ziyao Li, Yuji Wang, Jiejun He, Xiatian Ji, Huasheng Zhou, Xiaofan Zhang, Shigang Chen, Yezhen Zhou, Kaihui Xu, Guancong Liang, Haocheng Kuang, Yuting Gao, Xiaohua Liu, Luting Luo, Lin Ding, Yiji Li, Zhuanzhuan Liu, Tengfei Zhou, Zetian Lai, Xinghua Su, Yuyan Guo, Chenying Li, Lihua Xie, Minqing Li, Xinglong Wu, Jianhao Huang, Weicong Su, Yicheng Pan, Wei Hu, Dongrui Zhou, Chunv Li, Juan Gui, Jiazhi Ma, Xiaoli Feng, Minyi Zhu, Shangbin Zhong, Fan Chen, Huanchao Zeng, Yingxian Wu, Chen Wang, Shukai Li, Qi Wang, Xueyi Wang, Yekai Zhou, Jianxun Ling, Yingjie Liu, Shang Wu, Zhiwei Li, Minghui Zhong, Wenxia Wei, Lixian Xie, Xianli Xu, Hehai Huang, Guilan Yang, Yan Liu, Siting Liang, Yingxia Wu, Deyu Zhang, Changqing Xu, Jie Wang, Chunmei Wang, Rangke Wu, Zhicong Yang, Xiao-Guang Chen, Xiaohong Zhou

**Affiliations:** 1 Department of Pathogen Biology, Institute of Tropical Medicine, Key Laboratory of Prevention and Control for Emerging Infectious Diseases of Guangdong Higher Institutes, Guangdong Provincial Key Laboratory of Tropical Disease Research, School of Public Health, Southern Medical University, Guangzhou, 510515 China; 2 Guangzhou Center for Disease Control and Prevention, Guangzhou, 510440 China; 3 State Key Laboratory of Organ Failure Research, Department of Biostatistics, Guangdong Provincial Key Laboratory of Tropical Disease Research, School of Public Health, Southern Medical University, Guangzhou, 510515 China; 4 Conghua District Center for Disease Control and Prevention, Guangzhou, 510900 China; 5 Department of Landscape Architecture and Regional & Community Planning, College of Architecture, Planning and Design, Kansas State University, Manhattan, KS 66506 USA; 6 The School of Foreign Studies, Southern Medical University, Guangzhou, 510515 China

**Keywords:** *Aedes albopictus*, Habitat-preferences, Ecological heterogeneity, Urbanization levels, Land use categories, Seasonal variations, Climatic variables, Overwintering diapause eggs, Community-based intervention, Sustainable vector management

## Abstract

**Background:**

*Aedes albopictus* is an invasive vector of serious *Aedes*-borne diseases of global concern. Habitat management remains a critical factor for establishing a cost-effective systematic strategy for sustainable vector control. However, the community-based characteristics of *Ae. albopictus* habitats in complex urbanization ecosystems are still not well understood.

**Methods:**

A large-scale investigation of aquatic habitats, involving 12 sites selected as representative of four land use categories at three urbanization levels, was performed in Guangzhou, China during 2015–2017. The characteristics and dynamics of these *Ae. albopictus* habitats were assessed using habitat-type composition, habitat preference, diversity indexes and the Route index (RI), and the temporal patterns of these indexes were evaluated by locally weighted scatterplot smoothing models. The associations of RI with urbanization levels, land use categories and climatic variables were inferred using generalized additive mixed models.

**Results:**

A total of 1994 potential habitats and 474 *Ae. albopictus*-positive habitats were inspected. The majority of these habitats were container-type habitats, with *Ae. albopictus* showing a particularly higher habitat preference for plastic containers, metal containers and ceramic vessels. Unexpectedly, some non-container-type habitats, especially ornamental ponds and surface water, were found to have fairly high *Ae. albopictus* positivity rates. Regarding habitats, the land use category residential and rural in Jiangpu (Conghua District, Guangzhou) had the highest number of *Ae. albopictus* habitats with the highest positive rates. The type diversity of total habitats (H-total) showed a quick increase from February to April and peaked in April, while the H-total of positive habitats (H-positive) and RIs peaked in May. RIs mainly increased with the monthly average daily mean temperature and monthly cumulative rainfall. We also observed the accumulation of diapause eggs in the winter and diapause termination in the following March.

**Conclusions:**

Ecological heterogeneity of habitat preferences of *Ae. albopictus* was demonstrated in four land use categories at three urbanization levels. The results reveal diversified habitat-type compositions and significant seasonal variations, indicating an ongoing adaptation of *Ae. albopictus* to the urbanization ecosystem. H-positivity and RIs were inferred as affected by climatic variables and diapause behavior of *Ae. albopictus*, suggesting that an effective control of overwintering diapause eggs is crucial. Our findings lay a foundation for establishing a stratified systematic management strategy of *Ae. albopictus* habitats in cities that is expected to complement and improve community-based interventions and sustainable vector management.

**Graphical Abstract:**

**Figure:**
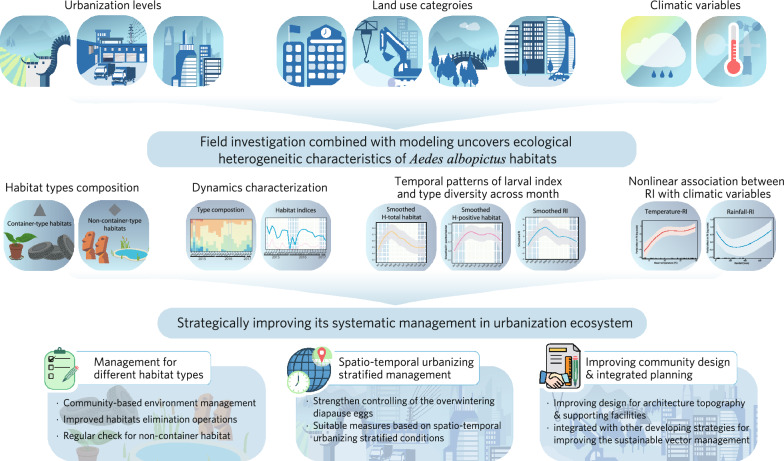

**Supplementary Information:**

The online version contains supplementary material available at 10.1186/s13071-023-05926-7.

## Background

*Aedes albopictus* ranks among the top 100 invasive species worldwide [1–3], and further expansion has been reported along the fringes of its current distribution [4]. This mosquito species is native to tropical Southeast Asia islands of the West Pacific and the Indian Ocean, but it has spread and colonized areas in all continents except Antarctica, across temperate, subtropical and tropical regions [5]. *Aedes albopictus* is a competent vector for several diseases of global concern, such as dengue, Zika virus disease and Chikungunya. Approximately 390 million dengue virus (DENV) infections are estimated to occur annually across the globe [6]. In China, dengue cases have been reported each year since 1990, and *Ae. albopictus* is the main vector of DENV [7, 8]. Also worrisome, is the major health threat worldwide posed by Zika virus disease, another *Aedes*-borne disease, after its pandemic in South America in 2015 [9].

Vector control remains the most crucial and effective method for blocking the transmission of *Aedes*-borne diseases. Insecticide-based strategies are the mainstay of vector mosquito control strategies, including long-lasting insecticide-treated nets (LLINs), indoor residual spraying (IRS), aerial sprays and insecticide-treated traps [10, 11]. However, insect populations are developing resistance to insecticides at an alarming rate, which is driving the search for new strategies to reduce the transmission of vector-borne diseases [12, 13]. Various new strategies have been developed, including those targeting vector population suppression and/or replacement, such as the radiation-based sterile insect technique (SIT), *Wolbachia*-based incompatible insect technique (IIT), entomopathogenic fungi (*Beauvaria bassiana* and *Metarhizium anisopliae*), sterility-inducing gene drives and pathogen-blocking gene drives [11, 14–19]. However, these techniques still require optimization by way of performing considerable testing for further assessment and improvement of their cost-effectiveness as well as engaging their roles in the environment from the prospective of safety.

Although all of above control strategies and techniques are available, habitat management in response to the ecological characteristics of the vector, as a long-established, traditional approach involving the participation of communities, still plays a critical role in the fight against vector-borne diseases. One such example is the program aimed at eliminating schistosomiasis through environmental measures directed towards eliminating the snail vector (*Oncomelania hupensis*) of the causal pathogen *Schistosomiasis japonica*. The program initiated in China has been an inspiring success, with measures directed towards the modification and elimination of *O. hupensis* habitats by way of reclaiming wetlands, digging new ditches and filling the old ones and changing rice paddies into dry fields, in parallel with other integrated interventions, such as molluscicide treatment [20]. Such active community-based interventions have also proven to be valuable and effective in mass campaigns of tackling the COVID-19 pandemic in the last 3 years [21, 22]. In terms of *Aedes* vectors, impressively, a large-scale program on mosquito habitats management has been implemented by the Patriotic Health Campaign System (PHCS) of China since 1978, with multiple achievements. During 1978–1991, for example, the Integrated Vector Management (IVM) on *Ae. aegypti* was initiated with the aim to block serious dengue transmission in Hainan Province of China. The main* Aedes* habitats control policies including "turning the basins and pots upside down" were proposed based on the systematic investigation of *Ae. aegypti* ecological habits in Hainan, which may have contributed to some extent to a greatly reduced number of reported dengue outbreaks in Hainan over the past two decades [23, 24]. However, in recent years, concomitant with climate warming, rapid globalization and urbanization, dengue outbreaks mainly transmitted by *Ae. albopictus* have occurred consecutively in the southern regions in China, with a tendentious spread to its northwest regions. Moreover, *Ae. albopictus* behaviors are not quite clear from the bio-ecological perspective, especially in the urbanized ecosystem. This pending issue poses challenges for the systematic management of *Ae. albopictus* habitats in the urbanized areas, especially at the community level in modern cities. Thus, an exploration of this aspect is urgent.

Urbanization involves the conversion of land use from rural uses to urban uses, such as cities where large and dense populations live and engage in secondary and tertiary industries. The urbanization ecosystem provides a large number of habitats for the dengue vectors, which show potent adaptability. For example, Guangzhou of China; the average growth rate of its urban area from 1987 to 2015 was 38.72 km^2^ per year [25], which undoubtedly facilitated the spreading of mosquito-borne diseases [26, 27]. Predominant among the wide range of mosquito species in the natural environment, *Ae. albopictus* is well adapted to urbanization ecosystems [28]. It grows and develops preferably in urban environments, where its larval developmental rate is remarkably increased and its adult survival time is significantly lengthened [26]. Abundant blood sources in urban environments also drives the blood-feeding behavior of this mosquito species to prefer the blood from humans [29]. One of the more beneficial factors, however, is the significant reduction in the variety and number of their predators. This, coupled with the urbanization-induced loss of inter-species diversity, hinders the inherent regulation and limitation of *Aedes* population density. *Aedes albopictus* is the sole vector of DENV, resulting in frequent epidemics of dengue across Guangzhou. In the large dengue outbreak in 2014, the number of dengue cases reached close to 37,376 [30], with no indication of the presence of *Ae. aegypti* [31]. However, very few studies have been done to observe the actual effect of urbanization at the urban, suburban and rural levels on the ecological characteristics of *Ae. albopictus* habitats during rapid urbanization.

*Aedes* prefers to lay its eggs in small bodies of water, especially in containers, which allows it to be more adaptive to the urbanized ecosystem. Urbanization leads to land use heterogeneity, resulting in spatially fine-grained differences in bio-diversity patterns and their associated ecosystem services. Consequently, urbanization contributes to the ecological heterogeneity of the potential aquatic habitats of *Ae. albopictus*. The aquatic habitats of *Ae. albopictus* are highly diverse [26, 32–37], but results from systematic research aimed at revealing its habitat composition, seasonal variations and influencing factors are unclear, especially in terms of improving cost-effective management programs in growing cities. In recent years, we performed a large-scale field survey combined with laboratory experiments and mathematical modeling base on the subtropical environments of Guangzhou, a megacity located in the Guangdong-Hong Kong-Macao Greater Bay Area in China, systematically observing and revealing the bio-ecological characteristics of *Ae. albopictus* behaviors, including its photoperiodic diapause and host-seeking behavior [13, 38, 39]. Based on these findings, we launched the large-scale field investigation described in this article, further characterizing the aquatic habitats of *Ae. albopictus* wild populations in four land use categories at three urbanization levels for the purpose of improving vector control strategies.

## Methods

### Study area and sampling sites

Similar to our previous studies [13, 38, 39], this study is part of the Monitoring and Investigation of *Ae. albopictus* Wild Population program and the field experiments were conducted during 2015–2017 in Guangzhou, China. Sanyuanli (SYL) in Yuexiu District, Jiahe (JH) in Baiyun District and Jiangpu (JP) in Conghua District in Guangzhou were chosen as the study areas, as in our previous work [38], representing three urbanization levels: urban (SYL), suburban (JH) and rural (JP) settings, respectively. Sanyuanli is an old urban area with a population density of approximately 10,000/km^2^; its main land use types are residential and commercial building. Jiahe is a suburban area near the urban boundary of Baiyun Mountains; it features a mixture of residential buildings, construction sites, manufacturing facilities and parks and has a population density of approximately 3000/km^2^. Jiangpu is a rural area with a population density of < 500/km^2^; its main land use types are farmland and woodland. Each setting included four land use categories: park (PAR), residential area (RES), construction site (CON), and school (SCH). Thus, a total of 12 investigation sites were included in the study (Additional file 1: Figure S1).

### Aquatic habitat surveys, mosquito larvae sampling and identification

Based on the characteristics of *Ae. albopictus* habitats, we designed and performed surveys for investigating immature mosquitoes once a month from March 2015 to February 2017. Three teams, each comprising six well-trained personnel, performed each survey, following the plan survey route in the study areas. For the outdoor environment, all properties within the site (i.e. residential, abandoned, commercial and public services, greenbelt, cropland) and alleyways were surveyed, except for parcels whose owners refused access or places that were inaccessible due to physical barriers (e.g. fallen structures, active CON site). With the help of the local Centers for Disease Control and Prevention (Conghua District, Baiyun District and Yuexiu District), we obtained consent from the residents after a detailed explanation of the study purpose. We then inspected the indoor environment and surrounding areas for aquatic habitats of *Ae. albopictus*.

Aquatic habitats were determined as *Ae. albopictus*-positive habitats depending on the presence of larvae/pupae/eggs of *Ae. albopictus*, which were identified via the species identification key of Yin et al. [33]. Briefly, larval sampling was conducted using a standard dipping method. Once dipped, larvae and pupae were collected using a pipette, and individual numbers were counted. The samples were then transported to the laboratory where they were reared until emergence for species identification. Frozen mosquitoes were placed on a piece of white filter paper in a petri dish on a chill table, and the species was identified morphologically using taxonomic keys [40]. Occasionally a small number of *Culex quinquefasciatus* were identified coexisting with *Ae. albopictus* in the same breeding water; in this case, we also counted the habitat as an *Ae. albopictus-*positive habitat.

Each person participating in the survey followed the same criterion of habitat determination and categorization. Various types of small water bodies preferred by *Ae. albopictus*, as shown in Additional file 2: Figure S2, were assessed as potential habitats. Surveys of all potential habitats and positive habitats in one investigation site were performed for 2 h or until all aquatic habitats had been assessed. The location and the physical characteristics of the aquatic habitats and *Ae. albopictus-*positive habitats were recorded. The natural status of habitats in the investigation sites were not modified in any way during the course of our observations and surveys. The habitat preferences of *Ae. albopictus* were assessed using the aquatic habitat positivity as follows:: aquatic habitat positive rate = number of *Ae. albopictus-*positive habitats/total number of habitats.

### Diapause determination of overwintering eggs in container-type habitats

During the surveys, we gently brushed overwintering eggs off the inside of the dry containers that harbored *Aede*s larvae and pupae between November and March and collected these. Diapause determination of the collected eggs was performed as described in our previous study: (i) collected eggs were stimulated to hatch by completely submerging individual egg papers in water in a fresh petri dish and then re-drying them, after which the still unhatched eggs were again stimulated to hatch after 1 week; (ii) each egg paper that had been stimulated twice was placed in a new petri dish and bleached with Trpiš solution [41]; (iii) after bleaching for 30 min, the eggs were observed and counted under a stereomicroscope, and embryos of unhatched eggs that presented pigmented ocelli and an egg burster and lacked abnormal pigmentation or malformation were considered to be in diapause. The incidence of diapause was calculated as follows: diapause incidence (%) = (embryonated unhatched eggs × 100)/(embryonated unhatched eggs + hatched eggs) [13, 38, 39].

### Dynamics of *Ae. albopictus* larval index

The larvae population density of *Ae. albopictus* was estimated using a larval index, namely the Route index (RI; the number of positive containers per kilometer of inspection route) [42]. During the aquatic habitat surveys, we used the Android app “GPS tools” developed by Shenzhen Xiaolang Information Technology Co. Ltd (Shenzhen, China) to record the survey route distance. The habitat investigators followed the same survey route to eliminate bias in route selection. RI was calculated as follows: RI = (number of positive habitats)/(survey route distance) × 100%.

### Diversity of *Ae. albopictus* habitat types

Habitat type diversity index was calculated using the Shannon (H) index as follows: $H = - \sum {p_{i} } log_{e} p_{i}$, where *p*_*i*_ = fraction of the entire population made up of units (*i*) [43, 44]. The H index takes into consideration the number of habitats as well as the number of habitat types, such that communities with fewer habitats yield values closer to 0, while communities with many habitats yield values closer to 1. The H-total indicated the type diversity of the total potential habitats, and the H-positive represented the type diversity of positive habitats.

### Statistical analysis

All statistical analyses were conducted using R 4.1.2 (R Foundation for Statistical Computing, Vienna, Austria). The habitat type distribution was assessed by goodness of fit Chi-squared test (*χ*^2^) test. Differences in the proportion of habitat types between groups were assessed using Pearson’s *χ*^2^ test and Bayesian Pearson’s *χ*^2^ test. The effect size was estimated using Cramer’s *V*. Differences in H-total, H-positive and RI were assessed using Welch’s one-way analysis of variance, and *P* values were adjusted using Game-Howell test. Two-sided *P* < 0.05 was considered to indicate statistical significance.

### Dynamics characterization of *Ae. albopictus* habitats

Locally weighted scatterplot smoothing (LOESS) models were used to assess the temporal patterns of the time-series of H-total, H-positive, and RI across month. The 95% confidence interval of these three indexes was estimated by a bootstrap method.

### Associations of RI with urbanization level, land use category and climatic variables

We applied generalized additive mixed models to assess the associations of RI with urbanization level, land use category and climatic variables including the monthly average daily mean temperature and the monthly cumulative rainfall. Climatic variables were downloaded from the Guangzhou Climate Data Network (http://www.tqyb.com.cn/). Here, we used quasi-Poisson family adjusting for overdispersion in the number of positive habitats. The model is as follows:

$$\mathrm{log}\left(E\left[{Y}_{i,t}\right]\right)=\alpha +offset\left(\mathrm{log}\left[{Route}_{i,t}\right]\right)+{Year}_{t}+{Month}_{t}$$

$$+s\left({Temp}_{i},df=3\right)+s\left({Rain}_{i},df=3\right)+{{\beta }_{1}Urban}_{i}+{{\beta }_{2}Land}_{i}+{\alpha }_{i}$$,

where ${Y}_{i,t}$ and ${Route}_{i,t}$ are the observed number of positive habitats and inspection route in the sampling site *i* at time point *t*, respectively; ${Year}_{t}$ is a categorical variable of the calendar year, while ${Month}_{t}$ is the calendar month at time point *t*; $s(.)$ represents the cubic regression spline; $df$ stands for degree of freedom; ${Temp}_{i}$ and ${Rain}_{i}$ are the monthly average daily mean temperature and the monthly cumulative rainfall of sampling site *i*, respectively; ${Urban}_{i}$ and ${Land}_{i}$ represent urbanization level, and land use category of the sampling site *i*; $\mathrm{\alpha }$, ${\beta }_{1}$ and ${\beta }_{2}$ are regression coefficients for the intercept, urbanization level and land use category, respectively. ${\alpha }_{i}$ is a random-effect intercept for the sampling sites. We used the first-order autoregressive (AR1) structure to account for the temporal autocorrelation within a sampling site if necessary. The results of models are presented fitted with the data including and excluding the information collected in March for comparison, since numerous diapause eggs of *Ae. albopictus* hatching under suitable environments each March in Guangzhou directly result a rapid unusual increase of RI in March.

Sensitivity analyses were conducted to examine the robustness of the estimates of the associations of RI with urbanization level, land use category and climatic variables by: (i) including the monthly averages of daily minimum and maximum temperature instead of mean temperature in the model; and (ii) applying a quadratic function to calendar month.

## Results

### Habitat preferences of *Ae. albopictus*

**Fig. 1:**
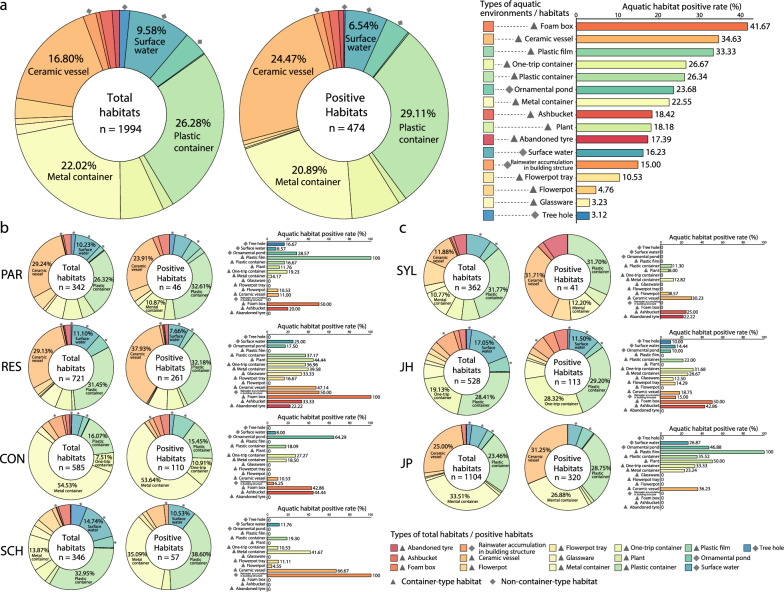
Ecological characteristics of *Aedes albopictus* habitats. **a** Type composition of 1994 potential total habitats, 474 *Ae. albopictus*-positive habitats and positive rate of different aquatic habitat types**. b** Ecological characteristics of *Ae. albopictus* habitats in different land use categories (CON, construction site; PAR, park; RES, residential area; SCH, school). **c** Ecological characteristics of *Ae. albopictus* habitats in different urbanization levels (JH, Jiahe [suburban]; JP, Jiangpu [rural]; SYL, Sanyuanli [urban]).

A total of 16 habitat types with 1994 potential habitats and 474 positive habitats were inspected at the 12 investigation sites, presenting a wide variety of aquatic habitats (Fig. 1a; Additional file 2: Figure S2). There were significant differences in the compositions of the types of total habitats (*χ*^2^ = 3230.75, *P* < 0.0001), positive habitats (*χ*^2^ = 1047.01, *P* < 0.0001) and the interaction between them (*χ*^2^ = 42.76, *P* < 0.0001;$\widehat{V}$=0.11; log_e_(BF_01_) = 7.47, $\widehat{V}$ = 0.14), indicating the heterogeneity of habitat types and the different habitat preferences of *Ae. albopictus* (Fig. 1a, Additional file 2: Figure S2). The majority of total habitats (93.0%, 1655/1994) and *Ae. albopictus*-positive habitats (88.2%, 418/474) were container-type habitats. Of these, plastic containers, metal containers and ceramic vessels accounted for the most abundant total and positive habitats, with positivity rates > 22%, implying the importance of these habitats for the management of *Ae. albopictus* (Fig. 1a). The surface water habitat also presented with a high positive rate of 16.23%, which was the highest among the non-container-type habitats (Fig. 1a; Additional file 2: Figure S2).

Surprisingly, we also observed non-container-type aquatic habitats with fitting positive rates in our survey. Many larvae and pupae of *Ae. albopictus* were found in unmanaged ornamental ponds in RES, in landscape pools with no fish, in stagnant waters in hidden corners of buildings and even in sculptures in SCH (Additional file 2: Figure S2). These habitats usually retained sufficient rainwater to be suitable breeding sites, especially during the rainy season (Additional file 2: Figure S2). We found that stacked paint buckets in the reclamation depots harbored large quantities of larvae and pupae of *Ae. albopictus* (Additional file 2: Figure S2).

### Ecological heterogeneity of habitat preferences of *Ae. albopictus* in different land use categories and urbanization levels

Regarding the different land use categories (RES, PAR, CON and SCH) (Fig. 1b, Additional file 3: Table S1) and urbanization levels (urban [SYL], suburban [JH] and rural [JP]) (Fig. 1c, Additional file 4: Table S2), significant differences of type compositions were found in total habitats, positive habitats and the interaction between them, indicating the ecological heterogeneity of habitat preferences of *Ae. albopictus*. However, regardless of land use category or urbanization level, *Ae. albopictus* showed the highest habitat preference for plastic containers, especially in RES and JP where the positive rates reached 37.17% and 35.52%, respectively (Fig. 1b).

**Fig. 2:**
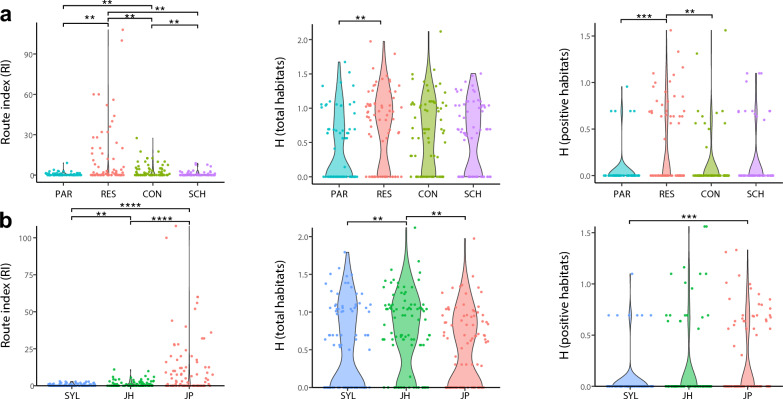
Respective comparison of RI, H-positive and H-total among four land use categories (CON, construction site; PAR, park; RES, residential area; SCH, school) (**a**) and three urbanization levels (JH, Jiahe [suburban]; JP, Jiangpu [rural]; SYL, Sanyuanli [urban]) (**b**). H-positive, Indicator of the type diversity of positive habitats; H-total, indicator of type diversity of the total potential habitats

Among the four land use categories, RES had the highest number of total habitats (721) and positive habitats (261), with the highest positivity rate of 36.2%, followed in decreasing order habitat preference by PAR (46/242, 19%), CON (110/585, 18.8%) and SCH (57/346, 16.5%) (Fig. 1b). The RI showed the consistent trend, with the RI of RES ($\widehat{\mu }$=12.24) higher than that of CON ($\widehat{\mu }$=3.26, *P* = 0.014), PAR ($\widehat{\mu }$=0.63, ρ_Holm-corrected_ = 0.014) and SCH ($\widehat{\mu }$=0.82, *P* = 0.014) (Fig. 2a). The H-total was also higher for RES ($\widehat{\mu }$=0.73) compared to PAR ($\widehat{\mu }$=0.40) and H-positive was higher for RES ($\widehat{\mu }$=0.32) compared to CON ($\widehat{\mu }$=0.11) and PAR ($\widehat{\mu }$=0.05) (Fig. 2a).

As shown in Fig. 1b, in RES, the top three habitats in terms of *Ae. albopictus* abundance were plastic containers of all types (227/721, 31.5%; 84/261, 32.2%), ceramic vessels (211/721 29.2%; 99/261, 37.9%) and the non-container-type surface waters (80/721, 11.1%; 20/261, 7.6%). Some habitat types, such as plastic containers, were found plentiful in all land use categories; accordingly, plastic containers were also abundant in the top habitat preferences in PAR (90/342, 26.3%; 15/46, 32.6%), CON (94/585, 16.1%; 17/110, 15.5%) and SCH (114/346, 33.0%; 22/57, 38.6%) (Fig. 1b). Other abundant habitat types, however, were specific to different land use categories; for example, ceramic vessels were more plentiful in RES and PAR, while metal containers were more plentiful in CON and SCH (Fig. 1b).

Among the three urbanization levels, the rural-JP site had the highest number of total habitats (1104) and positive habitats (320), with the highest positive rate of 29.0%, compared to the suburban-JH (113/528, 21.4%) and the urban-SYL (41/362, 11.3%) sites (Fig. 1c). The RI of JP ($\widehat{\mu }$=10.97) was higher than that of SYL ($\widehat{\mu }$=1.28, *P* < 0.001) and JH ($\widehat{\mu }$=0.46, *P* < 0.001). The H-total of JH ($\widehat{\mu }$=0.74) was higher than that of JP ($\widehat{\mu }$=0.51, ρ < 0.017) and SYL ($\widehat{\mu }$=0.52, *P* < 0.029) (Fig. 2b). The H-positive of JP ($\widehat{\mu }$=0.16) was higher than that of SYL ($\widehat{\mu }$=0.04, *P* < 0.001) (Fig. 2b).

As shown in Fig. 1c, in JP, the top three habitat preferences in terms of abundance were the metal container types (320/1104, 33.5%; 86/320, 26.9%), ceramic vessels (276/1104, 25.0%; 100/320, 31.3%) and plastic container types (259/1104, 23.5%; 92/320, 28.8%). Similarly, plastic containers as preferred habitats were also abundant in SYL (115/362, 31.8%; 13/41, 31.7%) and JH (150/528, 28.4%; 33/113, 29.2%). Some abundant habitat types also exhibited specificity in the urbanization levels; for example the container-type ceramic vessel (43/362, 11.9%; 13/41, 31.7%) was present in abundance in SYL, while non-container-type surface water (90/528, 17.1%; 13/113, 11.5%) was abundant in JP.

### Characterization of the dynamics of aquatic habitats

**Fig. 3:**
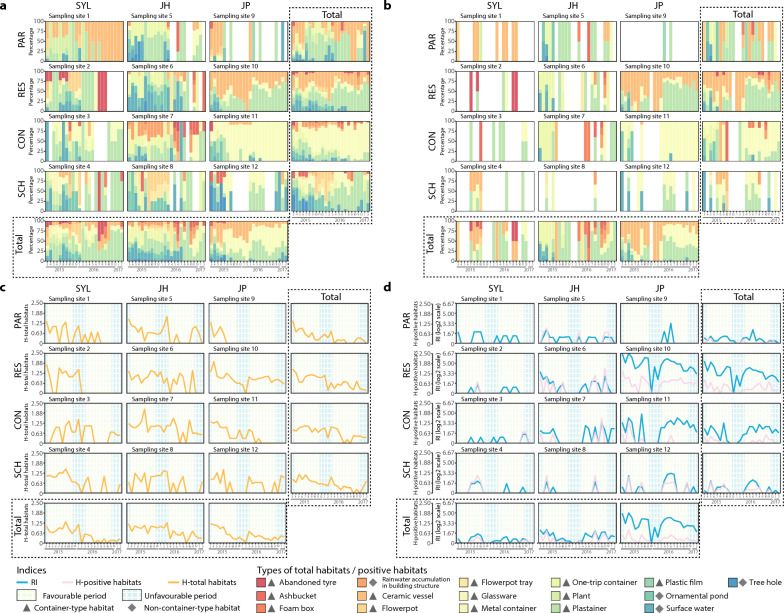
Monthly dynamics of type composition in total aquatic habitats (**a**) and positive habitats (**b**), and monthly variations of the H-total (**c**), H-positive and RI (**d**) of *Ae. albopictus* from March 2015 to February 2017 at four land use categories (CON, construction site; PAR, park; RES, residential area; SCH, school) in three urbanization levels (JH, Jiahe [suburban]; JP, Jiangpu [rural]; SYL, Sanyuanli [urban]) H-positive, Indicator of the type diversity of positive habitats; H-total, indicator of type diversity of the total potential habitats; RI, Route index

**Fig. 4:**
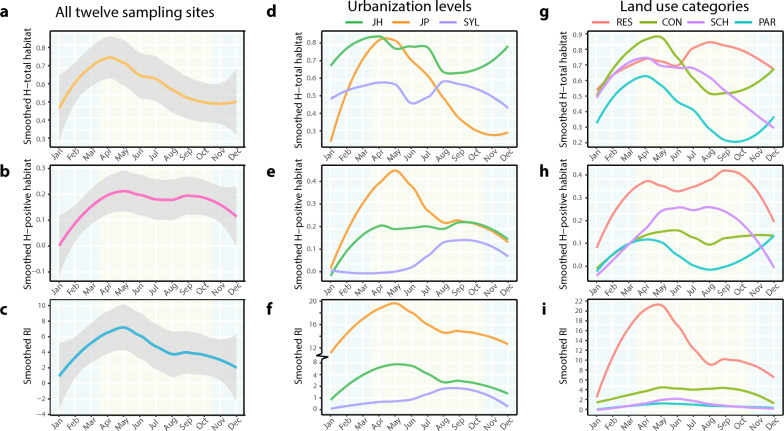
Temporal patterns of the smoothed H-total, H-positive, and RI fitting across months in all sampling sites (**a**, **b**, **c**), at three urbanization levels (JH, Jiahe [suburban]; JP, Jiangpu [rural]; SYL, Sanyuanli [urban]) (**d**,** e**,** f**) and at four land use categories (CON, construction site; PAR, park; RES, residential area; SCH, school) (**g**, **h**, **i**). H-positive, Indicator of the type diversity of positive habitats; H-total, indicator of type diversity of the total potential habitats; RI, Route index

The in-depth dynamics characterization of total aquatic habitats and positive habitats of *Ae. albopictus* also represented a significant ecological heterogeneity across months in the four land use categories at three urbanization levels in Guangzhou (Figs. 3, 4). Concretely, at all 12 sampling sites, the H-total, H-positive and RI, respectively, ranged from 0 to 2.11, from 0 to 1.56 and from 0 to 108, with mean values of 0.59 ± 0.47, 0.16 ± 0.25, and 4.20 ± 6.10, respectively (Fig. 3); these values indicate significant seasonal variations. The H-total rapidly increased from February to April and peaked in April, while both the the H-positive and RI peaked in May (Figs. 3, 4). After the May peak, the smoothed H-positive showed a continuous plateau, with another small peak in September and an obvious decline starting in November (Figs. 3, 4). According to the four land use categories, the H-total, H-positive and RI had the following ranges and mean values: 0 to 1.67, 0 to 0.96 and 0 to 9, respectively (mean values 0.40 ± 0.49, 0.05 ± 0.19 and 12.25 ± 22.29, respectively) in PAR; 0 to 1.97, 0 to 1.56 and 0 to 108, respectively (mean values 0.73 ± 0.55, 0.32 ± 0.43 and 0.68 ± 1.34, respectively) in RES; 0 to 2.12, 0 to 1.56 and 0 to 27.5, respectively (mean values 0.66 ± 0.52, 0.11 ± 0.30 and 3.27 ± 5.21, respectively) in CON; and 0 to 1.50, 0 to 1.10 and 0 to 8.8, respectively (mean values 0.58 ± 0.5, 0.14 ± 0.32 and 0.82 ± 1.88, respectively) in SCH (Figs. 3, 4). At the three urbanization levels, these three indexes had the following ranges and mean values: 0 to 1.79, 0 to 1.10 and 0 to 3, respectively (mean values 0.52 ± 0.55, 0.05 ± 0.20 and 0.47 ± 0.82, respectively) in SYL; 0 to 2.12, 0 to 1.56 and 0 to 11, respectively (mean values 0.73 ± 0.52, 0.17 ± 0.38 and 1.31 ± 2.11, respectively) in JH; and 0 to 1.97, 0 to 1.33 and 0 to 108, respectively (mean values 0.51 ± 0.50, 0.25 ± 0.37 and 10.97 ± 19.73, respectively), in JP (Fig. 3). Among thsem, the highest H-total of 2.11 occurred in sampling site 7 (JH-CON) in August 2015, the highest H-positive of 1.56 was found in sampling site 6 (JH-RES) in November 2016 and in sampling site 7 (JH-CON) in October 2016 and the highest RI of 108 was observed in sampling site 10 (JP-RES) in March 2015 (Fig. 3). The smoothed H-positive and RI were higher in JP and JH than in SYL. Among the four land use categories, higher indexes were observed in RES, especially the smoothed RI (Fig. 3, 4).

### Associations of RIs with urbanization levels, land use categories and climatic variables

The monthly averages of daily mean, minimum and maximum temperature ranged from 13.4 °C to 29.6 °C, from 9.7 °C to 25.4 °C and from 17.0 °C to 34.5 °C, and monthly cumulative rainfall ranged from 5.9 to 753.8 mm, with means of 22.8 °C, 19.3 °C, 27.3 °C and 194.5 mm, respectively. To conduct an in-depth exploration of the ecological heterogeneity mechanism of *Ae. albopictus* habitats, we applied generalized additive mixed models to infer the associations between RIs and urbanization levels, land use categories and climatic variables. Further modeling also indicated that the estimated relative risks of RIs in the rural-JP and the RES were higher than those in the other two urbanization levels and three land use categories, respectively (Table 1).

**Table 1:** Associations of the Route index with urbanization levels and land use categories inferred by generalized additive mixed models

Variable^a^	Based on all data		Excluding the data of March	
	RR	95% CI	RR	95% CI
*Urbanization levels*				
Rural-JP	Reference		Reference	
Suburban-JH	0.19	(0.07, 0.54)	0.17	(0.07, 0.43)
Urban-SYL	0.10	(0.03, 0.29)	0.08	(0.03, 0.23)
*Land use categories*				
RES	Reference		Reference	
CON	0.52	(0.15, 1.73)	0.53	(0.18, 1.50)
PAR	0.24	(0.07, 0.83)	0.23	(0.07, 0.70)
SCH	0.20	(0.06, 0.70)	0.22	(0.07, 0.65)

*CI*Confidence interval, *RR* relative risk

^a^JP, Jiangpu; JH, Jiahe; SYL, Sanyuanli; RES, residential area; CON, construction site; PAR, park; SCH, school

**Fig. 5:**
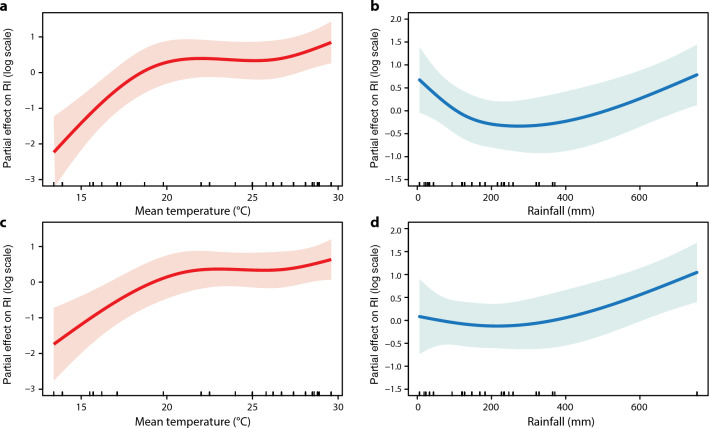
The exposure–response curves of the associations of RI with monthly average daily mean temperature (**a**, **c**) and monthly cumulative rainfall (**b**, **d**) from 12 sampling sites in Guangzhou. The exposure–response curves were based on all data (**a**, **b**) and excluding the data of March (**c**, **d**), respectively. The shaded areas represent the 95% confidence intervals of the estimates of partial effects. CON, Construction site; PAR, park; RES, residential area; RI, Route index; SCH, school

The exposure–response curves of RIs with the climatic variables indicated that the RI mainly increased with increasing monthly average daily mean temperature (Fig. 5), and also with the monthly minimum and maximum temperature (Additional file 6: Figure S3). The RIs increased rapidly with increasing monthly average daily mean temperature between 13.4 °C and 22.1 °C during the non-active period of *Ae. albopictus* populations from December to March, and then reached a plateau from 22.1 °C to 26.6 °C following a second slight rise from 26.6 °C to 29.6 °C, which was coincident with the active period of *Ae. albopictus* populations from April to November in Guangzhou (Fig. 5a, c). Based on the model fitted with all data, the RI curve associated with the monthly cumulative rainfall presented a slight U-shape, with its thresholds falling within the range 170–280 mm (Fig. 5b). Interestingly, excluding the data collected in March, we found that the RIs did not change substantially when monthly cumulative rainfall was less than approximately 300 mm, and that they mainly increased with increasing rainfall (Fig. 5d). The results of the sensitivity analyses showed that all estimates of the associations of RIs with urbanization levels (Additional file 5: Table S3), land use categories (Additional file 5: Table S3) and climatic variables (Additional file 6: Figure S3; Additional file 7: Figure S4) were robust.

### Diapause identification of overwintering eggs in container-type habitats

The photoperiodic diapause eggs were initially observed in the field in Guangzhou beginning in September, reaching a peak in the diapause rate of *Ae. albopictus* overwintering eggs around the winter solstice, with > 80% [38]. We also found that the dry containers harbored an abundance of accumulated diapause eggs in the winter, which could start to hatch into larvae under suitable environment in the following March, resulting in a rapid and unusual increase in the RI for this month (Additional file 8: Figure S5).

## Discussion

The results of our large-scale field survey demonstrate the highly ecological heterogeneity of *Ae. albopictus* habitats, whether container-type or non-container-type, indicating the ongoing adaptability of *Ae. albopictus* to the subtropical urbanization ecosystem in Guangzhou. Container-type habitats are thought to be crucial for the successful colonization and global dispersal of *Aedes* mosquito lineage, which may be the result of convergent evolution of ground-pool dwelling ancestors [45]. In our study, we observed that the wild populations of *Ae. albopictus* preferred container-type habitats, including plastic containers, metal containers, ceramic vessels and the single-use containers standardly used in Guangzhou. Many larvae and pupae of *Ae. albopictus* were also found in the non-container-type habitats, such as ponds and landscape pools with large water volumes, indicating the habitat expansion of *Ae. albopictus* during urbanization. Larvae/pupae of *Ae. albopictus* were commonly observed to be more abundant in a number of ponds or pools with a relatively larger water surface area, which may partly contribute to the increase in this mosquito’s population density in the urbanized ecosystem. This adaptability of *Ae. albopictus* to urbanized environments may be attributed to two potential factors: (i) natural predators, including dragonflies, bettas and frogs, are relatively rare in many of these artificial ponds or pools; and (ii) a short-distance fly contained in a limited space limits the mosquito’s immature stages to living in any type of fragmented aquatic habitat. Surprisingly, another non-container-type habitat, such as surface water, is also a concerning factor due to the high higher larvae/pupae positive rate observed in this study, especially during the rainy seasons; such surface water was commonly found in the land use categories of SCH, RES and PAR, and at the urbanization level in suburban JH in Guangzhou. The ideal *Ae. albopictus* breeding habitats should have plenty of food and no predators, with adult female mosquitoes having different preferences for habitat texture, color, illuminance intensity and organic and inorganic density, as well as for the bacterial microbiota in the habitat water [46]. As noted, *Ae. albopictus* had a high habitat preference plastic containers, mental containers, ceramic vessels, foam boxes and plastic film, indicating that these habitats types were suitable for larval development.

The aim of the present study was to systematically elucidate the heterogeneity of *Ae. albopictus* habitat types in the four land use categories at three urbanization levels. During the urbanization process, the factors influencing habitat heterogeneity are complex, with the primary factors being human behaviors, correct guidance to the public on vector control of mosquito-borne infectious diseases, effective management strategies and measures at the national level, climate warming and biological adaptation of *Ae. albopictus*, among others. Therefore, the compositions of the habitats varying among the four land use categories at the three urbanization levels. Limited by biological characteristics, such as flight ability, *Ae. albopictus *has gradually adapted and modified its habitat preference to special local environments. The habitat heterogeneity of *Ae. albopictus* is instructive for establishing a stratified management system. During the urbanization process of Guangzhou, the land use-RES and the rural-JP sites were characterized by a relatively more complicated ecosystem, abundant habitats, higher relative estimated risk of RIs and more dense *Ae. albopictus* populations, all factors of particular concern. To some extent, the suitability of the habitats for *Ae. albopictus* populations seem to be affected by differential application of management programs during urbanization. For example, a smaller number of positive habitats and a lower proportion of used containers may have a more positive effect on environmental management in PAR and SCH than RES and CON under local governance. From the perspective of urban villages (UVs) in RES, for example, great importance should be attached to dealing effectively with the diversified habitats of *Ae. albopictus*, including managing the disposable or waste containers left in grass or corners. In UVs, which are scattered across the well-urbanized downtown areas of Guangzhou, dengue incidence was reported to be 1.82- to 3.06-fold higher than in areas of normal construction land, with 90% of the total cases concentrated in a 500-m radius of UVs’ buffering zones; the main factor is the presence of UVs providing *Ae. albopictus* with suitable environments for breeding [47]. A survey of 11 construction sites in Miami-Dade County (Florida, USA) showed that the most productive mosquito habitats were elevator shafts, Jersey plastic barriers, flooded floors and stair shafts [48]. Interestingly, our findings showed that > 50% of the total and positive habitats of *Ae. albopictus* in the CONs in Guangzhou were metal containers. Different from results of Li et al. [26], who reported that the urban area presented an increased density of *Ae. albopictus*, our investigation showed that the rural-JP in Guangzhou had the highest abundance of total and positive habitats, possibly due to the different time points and survey sites selected for these two studies, as well as to improved urban governance in Guangzhou city in recent years [49].

We found that the ecological heterogeneity of *Ae. albopictus’s* habitat preferences varied significantly across seasons, a results which may have been complicated by inconsistent dynamics of total aquatic habitats' ecosystem, *Ae. albopictus* adult oviposition behavior and photoperiodic diapause egg hatching. The dynamics of *Ae. albopictus* population densities were demonstrated in our previous study [38]. Similarly, our temporal patterns of H-positive and RI of *Ae. albopictus* were found to rapidly increase from February to April, becoming higher during the favorable period from April to November and peaking in May; the lowest values were found for the unfavorable period from December to February. The RI also tended to fall to some extent from September at all three urbanization levels across the four land use categories; one inference is that this decline is related to the beginning of diapause induced by steady annual photoperiodic dynamics [38]. We observed that many diapause eggs were attached to the walls of dry containers in the winter and that these began to hatch in the following March. This finding implies that the H-positive and RIs may be strongly shaped by the diapause behavior of *Ae. albopictus*. The inference from our findings is consistent with the results of a previous study based on a mechanistic population model by Jia and colleagues, who inferred that diapause plays an important role in its population density dynamics in Guangzhou [50].

Based on our statistical model we also inferred a nonlinear but generally increasing association between RI and monthly average daily mean temperature, which is consistent with the findings of previous studies [51]. The fitted models based on all the data showed a slight U-shaped curve of exposure–response relationship between RI and monthly cumulative rainfall, suggesting a complex interaction between these two factors. Surprisingly, however, when the March data were excluded, RI was seen to predominantly increase with rainfall, further confirming that since diapause of *Ae. albopictus* began to terminate in March in Guangzhou, a large number of larvae incubated by overwintering diapause eggs directly led to the steep rise of RI, which obviously influenced and reshaped the correlation curve in the model.

The findings of our study emphasize the urgency and importance of proposing a better guidance for communities to participate in the control of overwintering diapause eggs by *Ae. albopictus* and the management of its habitats. One approach to controlling overwintering diapause eggs is to locate breeding sites, especially container-type habitats, even the dry ones, and then kill the eggs, which are mostly attached to the container walls, in the autumn and winter. Another crucial approach is to engage the PHCS to launch major campaigns in the Guangzhou region aimed at systematic control of habitats, beginning in March and continuing to the following spring. In terms of practically, the routine practices advised by the PHCS of China in such slogans as “turning the basins and pots upside down” to prevent or remove any accumulation of rain or sprinkler water in all containers, are effective in eliminating mosquito breeding sites. However, diapause or quiescent eggs are found to have certain resistance to dry or cold conditions [52]. This latest scenario on *Ae. albopictus*, based on an in-depth understanding of its diapause or quiescent eggs, teaches us a lesson that the traditional approaches need to be optimized to achieve control of the dengue vector, For example, if the eggs are laid or just dumped simply or directly into soil, incubation may only be delayed until the right conditions present themselves for hatching into larvae [38], especially in the rainy season when water may accumulate in the soil as it does in non-containerized surface waters, which have been found to test highly positive for *Ae. albopictus* larvae, as observed in the suburban JH and land use-SCH, RES and PAR in Guangzhou in our field investigations. For this reason, we recommend that the water in container-type habitats be diverted into the municipal sewers after the walls of the containers have been carefully cleaned and the eggs killed using integrative methods, such as hot water, heat and/or insecticides, especially in the rainy season.

**Fig. 6:**
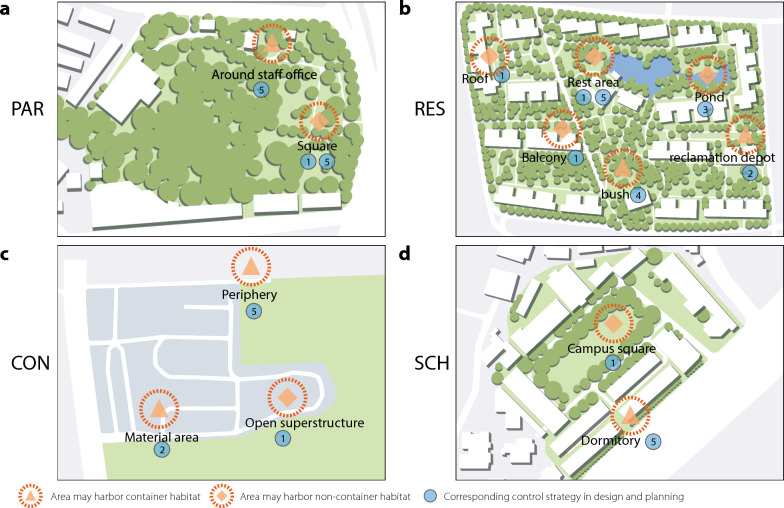
Guidance for improvement of landscape architecture and urbanizing community-based planning to control the presence of potential habitats of vector mosquitoes in four different land use categories (**a** CON, construction site; **b** PAR, park; **c** RES, residential area; **d** SCH, school) during urbanization

Active community engagement has proven to be one of the important strategies for tackling the COVID-19 pandemic [21, 22]. The establishment of a precisely stratified habitat management strategy that relies on community networks, based on our findings related to the ecological heterogeneity of *Ae. albopictus* habitats in different land-use categories at different levels of urbanization, would also be critical to interrupting the cycle of mosquito-borne diseases. Based on the distribution of container-type and non-container-type habitats in the four land use categories shown in Fig. 6, and referring to the view of landscape architecture and urbanized community planning, we propose five front-loading vector control recommendations that may be beneficial for future community-based integrated interventions of *Ae. albopictus*:

(i) Reduce the possibility of water accumulation in topography design. In this context, special attention should be paid to the choice of ornament shape on roofs and balconies and in open squares (including sculptures, art installations and rockeries etc.) in residential buildings. The drainage systems of residential areas should be carefully designed and managed so as to avoid the accumulation of rainwater. In designing such landscapes, storm water management can be improved through topography design, the use of permeable pavements and the appropriate selection of plant species (Fig. 6, ①).
(ii) Avoid designing open-air public facilities, such as reclamation depots for the community (Fig. 6, ②).
(iii) Use flowing water in artificial ponds and ensure that artificial ponds are correctly managed. (Fig. 6, ③).
(iv) Replace bushes with lawns, especially in residential areas used for outdoor activities (Fig. 6, ④).
(v) Provide effective facilities and feasible solutions for adequate mosquito adult control around residential rest areas in the land-use RES, campus squares and dormitory areas in SCH and in squares and staff office areas in PAR (Fig. 6, ⑤).

## Conclusion

In summary, the present field investigation and statistical modeling approach reveal the ecological heterogeneitic characteristics of *Ae. albopictus* habitats in four land use categories at three urbanization levels in Guangzhou. We found highly diversified habitat type compositions and seasonal variations in the diversity indexes and RIs. The H-positive and RIs rapidly increased from February to April and peaked in May, and RIs mainly increased with the average daily mean temperature and cumulative rainfalls on monthly basis. These findings imply that the H-positive and RIs are significantly affected by climatic variables, and that they are also clearly re-shaped by the diapause behavior of *Ae. albopictus*. Therefore, it is imperative to effectively control the overwintering diapause eggs in winter and the larvae hatched by diapause termination in the following March. It is also important to optimize operations aimed at eliminating favorable habitats, to explore suitable measures based on spatio-temporal urbanizing stratified conditions and to adapt suitable architecture styles in communities. Our findings also indicate an ongoing adaptability of *Ae. albopictus* in urbanized ecosystems, warning us of a high risk of the transmission of dengue or other *Aedes*-borne diseases. The present study lays a foundation for strategically improving a stratified systematic management of *Ae*. *albopictus* habitats. Hopefully, the stratified systematic management can also be integrated with other developing strategies for an improvement of community-based interventions and sustainable vector management.

### Supplementary Information

**Additional file 1: Figure S1.** Study areas and surrounding environments of the selected investigation sites in Guangzhou.** a** SYL (an urban area in Yuexiu District),** b** JH (a suburban area in Baiyun District),** c** JP (a rural area in Conghua District) represent the three urbanization levels. The green triangles, rhombus, square and pentagram indicate construction site (CON), park (PAR), residential area (RES) and school (SCH), respectively, that correspond to the four land use categories.

**Additional file 2: Figure S2.** A wide variety of aquatic habitats were inspected in the 12 study sites.

**Additional file 3: Table S1.** Equality and comparison of composition of total aquatic habitats and positive habitats in the four land use categories.

**Additional file 4: Table S2.** Equality and comparison of composition of total aquatic habitats and positive habitats in the three urbanization levels.

**Additional file 5: Table S3.** Associations of RI with urbanization levels and land use categories in the sensitivity analysis.

**Additional file 6: Figure S3.** Exposure-response curves of the associations of RI with temperature and rainfall in the sensitivity analysis. **a** The exposure-response curve of the association between RI and monthly average of daily minimum temperature based on the model with minimum temperature. **b** The exposure-response curve of the association between RI and monthly cumulative rainfall based on the model with minimum temperature. **c** The exposure-response curve of the association between RI and monthly average of daily maximum temperature based on the model with maximum temperature. **d** The exposure-response curve of the association between RI and monthly cumulative rainfall based on the model with maximum temperature. **e** The exposure-response curve of the association between RI and monthly average of daily mean temperature based on the model in which a quadratic function was applied for month. **f** The exposure-response curve of the association between RI and monthly cumulative rainfall based on the model in which a quadratic function was applied for month.

**Additional file 7: Figure S4.** Exposure-response curves of the associations of RI with temperature and rainfall when excluding the data of March in the sensitivity analysis. **a** The exposure-response curve of the association between RI and monthly average of daily minimum temperature based on the model with minimum temperature. **b** The exposure-response curve of the association between RI and monthly cumulative rainfall based on the model with minimum temperature. **c** The exposure-response curve of the association between RI and monthly average of daily maximum temperature based on the model with maximum temperature. **d** The exposure-response curve of the association between RI and monthly cumulative rainfall based on the model with maximum temperature. **e** The exposure-response curve of the association between RI and monthly average of daily mean temperature based on the model in which a quadratic function was applied for month. **f** The exposure-response curve of the association between RI and monthly cumulative rainfall based on the model in which a quadratic function was applied for month.

**Additional file 8: Figure S5.** Dry containers (**a**,** b**), overwintering eggs collection (**c**) and identified *Ae. albopictus* diapause eggs (**d**).

## Data Availability

The datasets used and/or analyzed during the current study are available from the corresponding author on reasonable request.

## Abbreviations

CON: Construction site
CrI: Credible intervals
DENV: Dengue virus
H: Shannon index
IIT: Incompatible insect technique
IRS: Indoor residual spraying
IVM: Integrated vector management
LLINs: Long-lasting insecticide-treated nets
PAR: Park
PHCS: Patriotic Health Campaign System
RES: Residential area
RI: Route index
SCH: School
SIT: Sterile insect technique
UVs: Urban villages
