# Supplementary figures and images for: Field investigation combined with modeling uncovers the ecological heterogeneity of Aedes albopictus habitats for strategically improving systematic management during urbanization
Source: Parasit Vectors. 2023 Oct 25;16:382. doi: 10.1186/s13071-023-05926-7 (PMC10599048; doi:10.1186/s13071-023-05926-7)

▲ PAR

● RES

■ CON

★ SCH

SYL

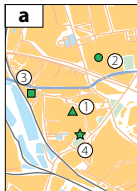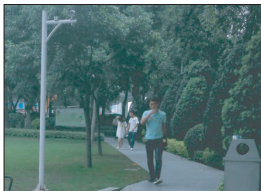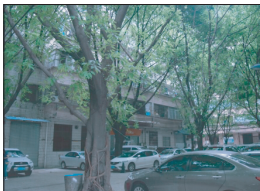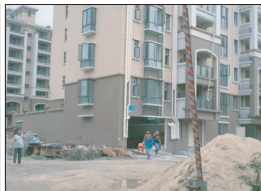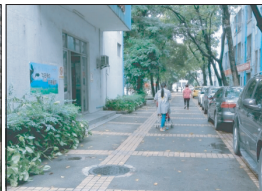

JH

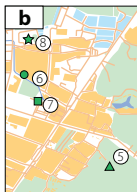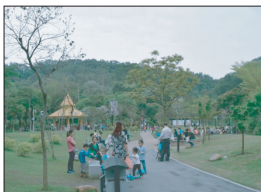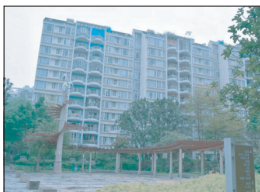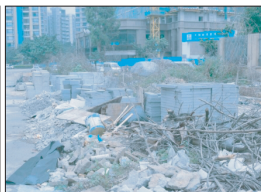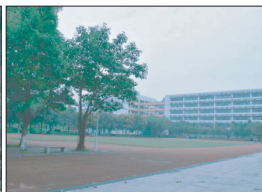

JP

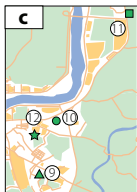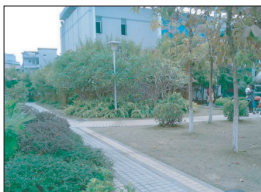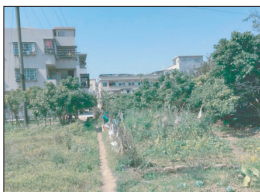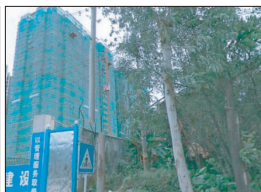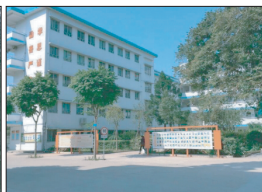

a: 400m b,c: 600m

Supplement: Supplementary file 1 — Additional file 1: Figure S1. Study areas and surrounding environments of the selected investigation sites in Guangzhou. a SYL (an urban area in Yuexiu District), b JH (a suburban area in Baiyun District), c JP (a rural area in Conghua District) represent the three urbanization levels. The green triangles, rhombus, square and pentagram indicate construction site (CON), park (PAR), residential area (RES) and school (SCH), respectively, that correspond to the four land use categories. [file 13071_2023_5926_MOESM1_ESM.pdf]

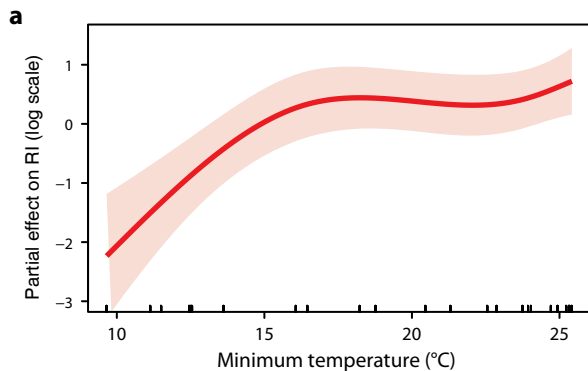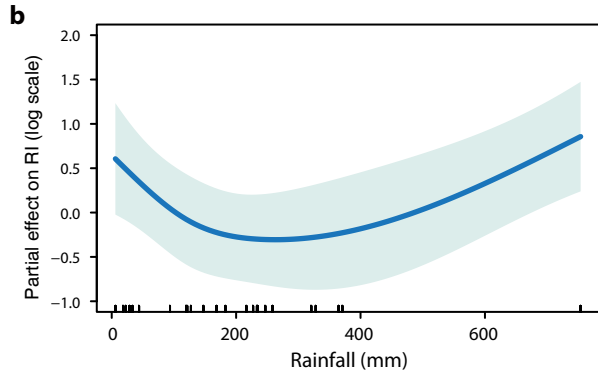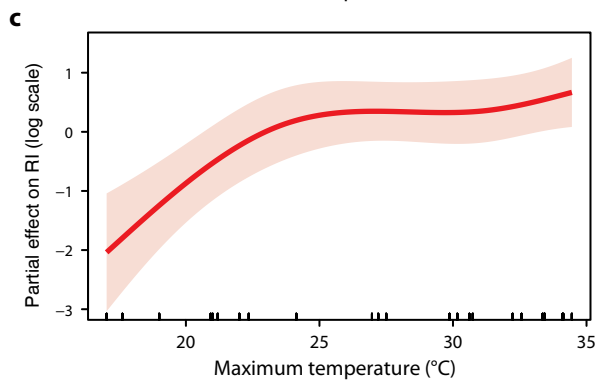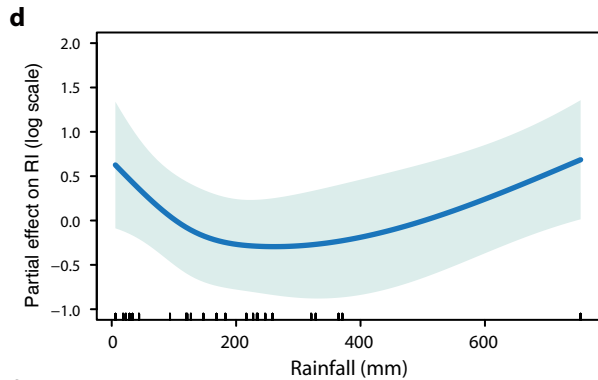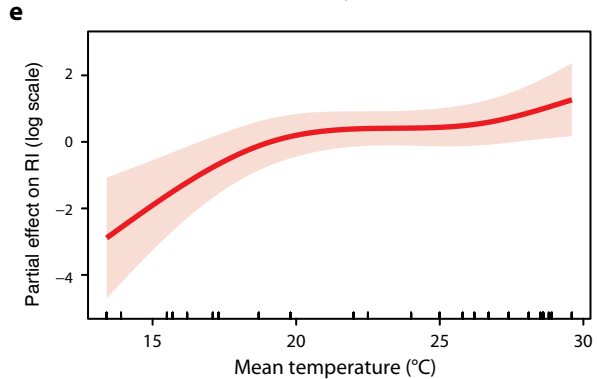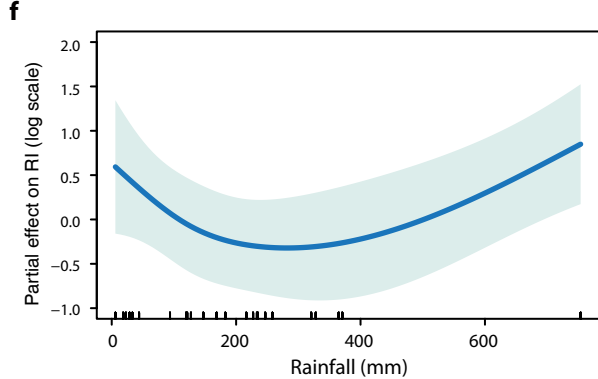

Supplement: Supplementary file 3 — Additional file 3: Table S1. Equality and comparison of composition of total aquatic habitats and positive habitats in the four land use categories. [file 13071_2023_5926_MOESM3_ESM.pdf]

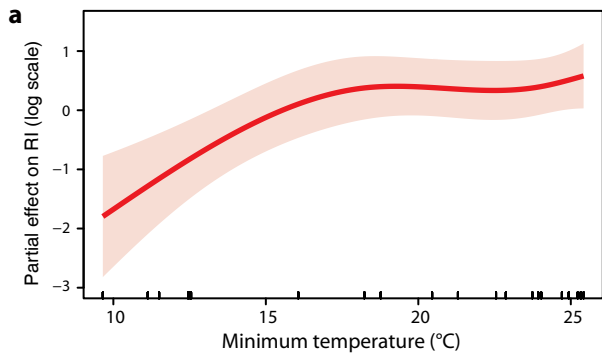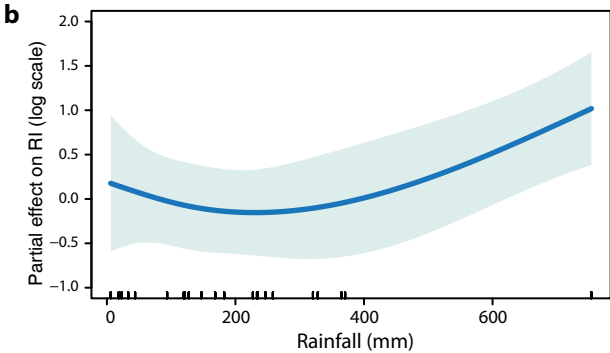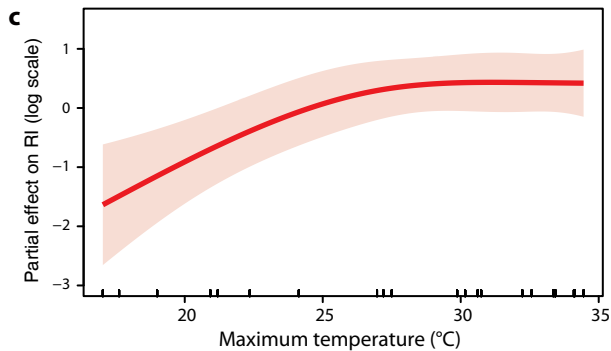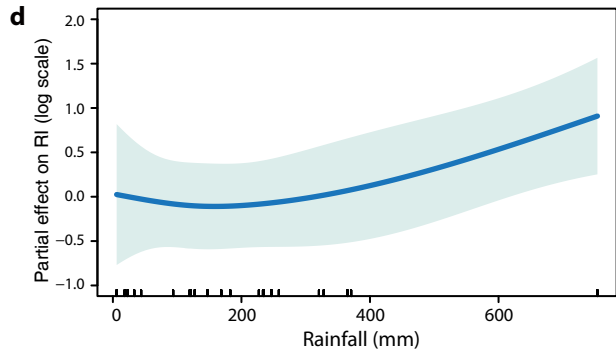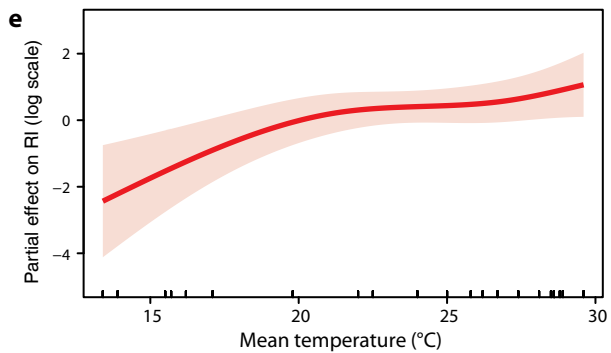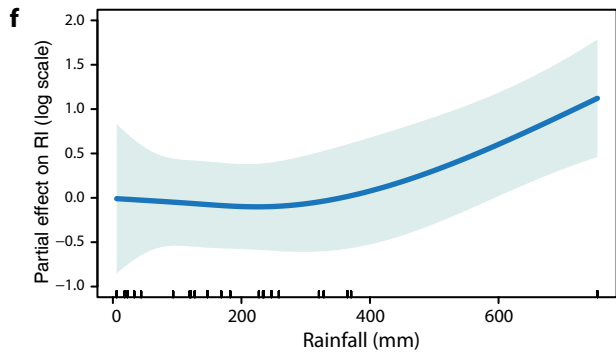

Supplement: Supplementary file 4 — Additional file 4: Table S2. Equality and comparison of composition of total aquatic habitats and positive habitats in the three urbanization levels. [file 13071_2023_5926_MOESM4_ESM.pdf]

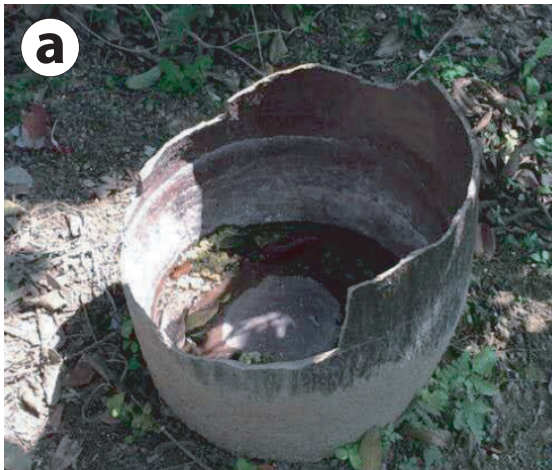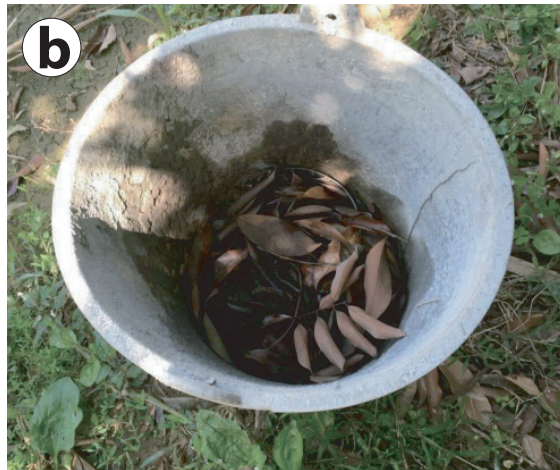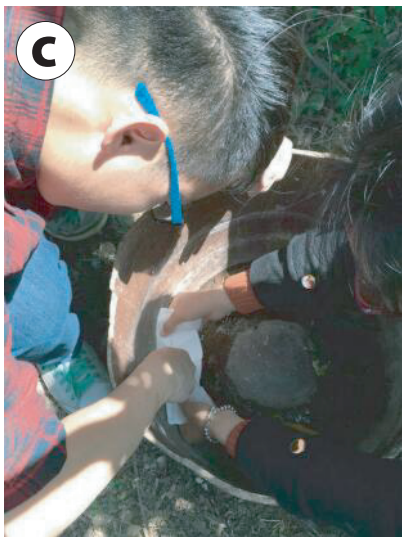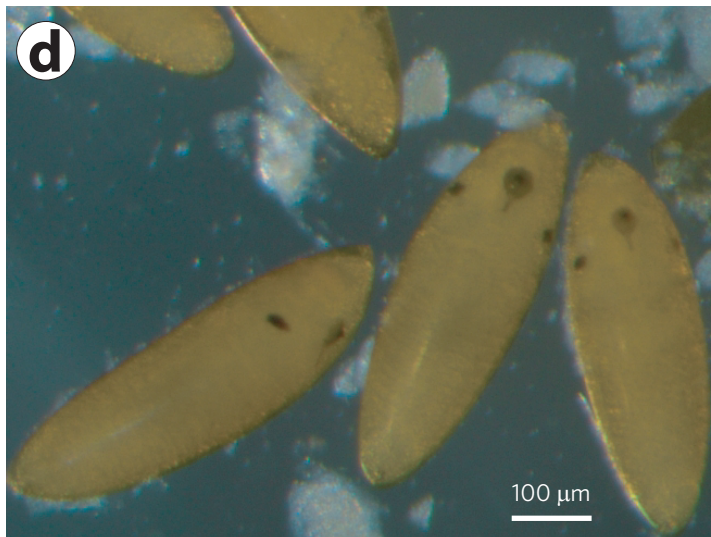

Supplement: Supplementary file 5 — Additional file 5: Table S3. Associations of RI with urbanization levels and land use categories in the sensitivity analysis. [file 13071_2023_5926_MOESM5_ESM.pdf]
