# Supplementary material for: Field investigation combined with modeling uncovers the ecological heterogeneity of Aedes albopictus habitats for strategically improving systematic management during urbanization
Source: Parasit Vectors. 2023 Oct 25;16:382. doi: 10.1186/s13071-023-05926-7 (PMC10599048; doi:10.1186/s13071-023-05926-7)
Supplement: Supplementary file 2 — Additional file 2: Figure S2. A wide variety of aquatic habitats were inspected in the 12 study sites. [file 13071_2023_5926_MOESM2_ESM.pdf]

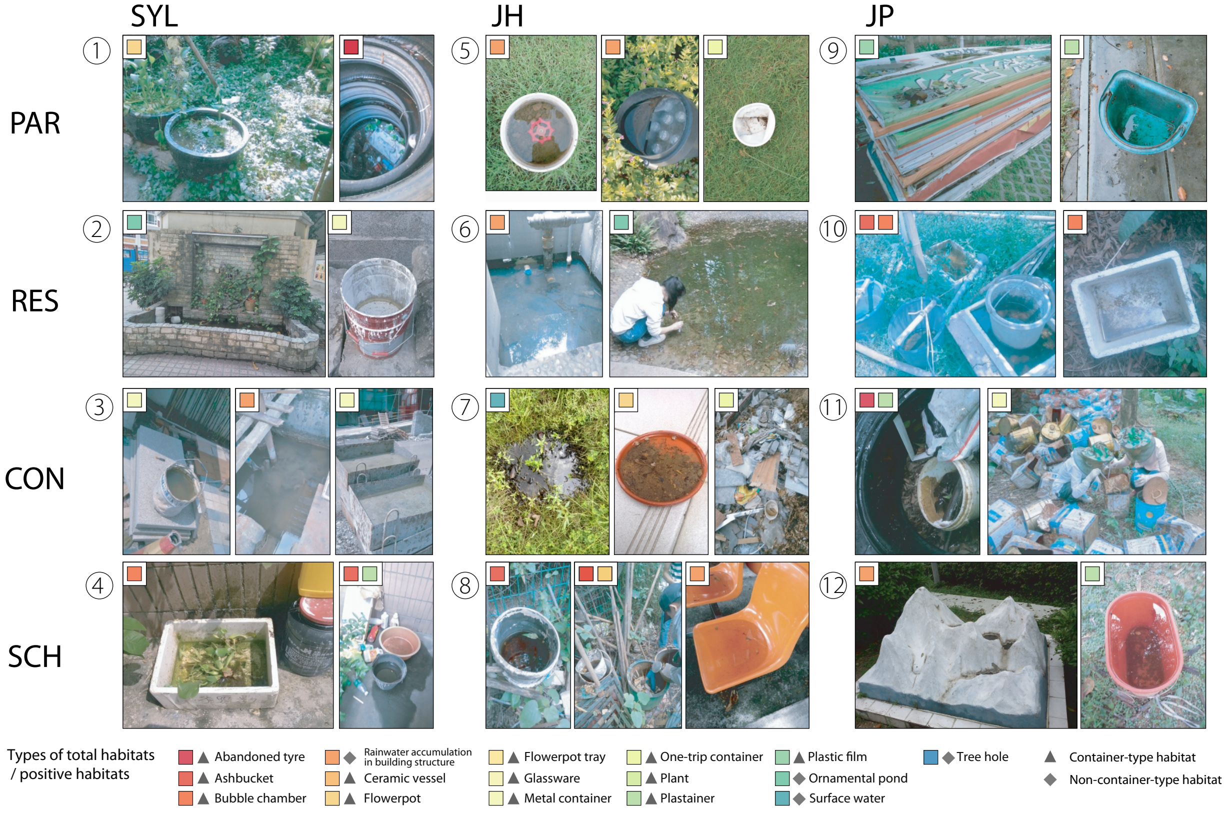

SYL
JH
JP
PAR
RES
CON
SCH
1
2
3
4
5
6
7
8
9
10
11
12
Types of total habitats / positive habitats
Abandoned tyre
Ashbucket
Bubble chamber
Rainwater accumulation in building structure
Ceramic vessel
Flowerpot
Flowerpot tray
Glassware
Metal container
One-trip container
Plant
Plastainer
Plastic film
Ornamental pond
Surface water
Tree hole
Container-type habitat
Non-container-type habitat
