# Supplementary material for: Field investigation combined with modeling uncovers the ecological heterogeneity of Aedes albopictus habitats for strategically improving systematic management during urbanization
Source: Parasit Vectors. 2023 Oct 25;16:382. doi: 10.1186/s13071-023-05926-7 (PMC10599048; doi:10.1186/s13071-023-05926-7)
Supplement: Supplementary file 7 — Additional file 7: Figure S4. Exposure-response curves of the associations of RI with temperature and rainfall when excluding the data of March in the sensitivity analysis. a The exposure-response curve of the association between RI and monthly average of daily minimum temperature based on the model with minimum temperature. b The exposure-response curve of the association between RI and monthly cumulative rainfall based on the model with minimum temperature. c The exposure-response curve of the association between RI and monthly average of daily maximum temperature based on the model with maximum temperature. d The exposure-response curve of the association between RI and monthly cumulative rainfall based on the model with maximum temperature. e The exposure-response curve of the association between RI and monthly average of daily mean temperature based on the model in which a quadratic function was applied for month. f The exposure-response curve of the association between RI and monthly cumulative rainfall based on the model in which a quadratic function was applied for month. [file 13071_2023_5926_MOESM7_ESM.docx]

**Table S2** Equality and comparison of compositions of total aquatic habitats and positive habitats in three urbanization levels

(1) Equality of compositions of total aquatic habitats and positive habitats in three urbanization levels

| Types | No | Variables | Pearson’s $$^2^ test | |
| --- | --- | --- | --- | --- |
|  |  |  | $_{gof}^{2}$ | *p* |
| Total habitat | (1) | Urban | 554.95 | <0.001 |
|  | (2) | Suburban | 842.97 | <0.001 |
|  | (3) | Town/rural | 3058.75 | <0.001 |
| Positive habitat | (1) | Urban | 112.37 | <0.001 |
|  | (2) | Suburban | 236.31 | <0.001 |
|  | (3) | Town/rural | 1002.10 | <0.001 |

(2) Comparison of compositions of total aquatic habitats and positive habitats in three urbanization levels

| Types | No | Variables | Pearson’s $$^2^ test | | | Bayesian Pearson’s $$^2^ test | |
| --- | --- | --- | --- | --- | --- | --- | --- |
|  |  |  | $_{Pearson}^{2}$ | *p* | $\hat{V}_{cramer}$ | log_e_(BF_01_) | $\hat{V}_{cramer}^{posterior}$ |
| Total habitat | (1) | Urban ~ Suburban ~ Town/rural | 857.09 | <0.001 | 0.46 | 361.45 | 0.46 |
|  | (2) | Urban ~ Suburban | 203.81 | <0.001 | 0.46 | 89.78 | 0.47 |
|  | (3) | Urban ~ Town/rural | 275.78 | <0.001 | 0.42 | 106.30 | 0.43 |
|  | (4) | Suburban ~ Town rural | 559.99 | <0.001 | 0.58 | 280.40 | 0.58 |
| Positive habitat | (1) | Urban ~ Suburban ~ Town/rural | 284.41 | <0.001 | 0.52 | 79.67 | 0.50 |
|  | (2) | Urban ~ Suburban | 73.64 | <0.001 | 0.62 | 21.64 | 0.59 |
|  | (3) | Urban ~ Town/rural | 73.40 | <0.001 | 0.42 | 7.64 | 0.43 |
|  | (4) | Suburban ~ Town rural | 183.48 | <0.001 | 0.63 | 73.00 | 0.62 |
